# Generating Spatial Referring Expressions in a Social Robot: Dynamic vs. Non-ambiguous

**DOI:** 10.3389/frobt.2019.00067

**Published:** 2019-08-02

**Authors:** Christopher D. Wallbridge, Séverin Lemaignan, Emmanuel Senft, Tony Belpaeme

**Affiliations:** ^1^CRNS, School of Computing, Electronics and Mathematics, University of Plymouth, Plymouth, United Kingdom; ^2^Bristol Robotics Laboratory, University of West England, Bristol, United Kingdom; ^3^IDlab - imec, Ghent University, Ghent, Belgium

**Keywords:** Human Robot Interaction, natural language, spatial referring expressions, dynamic description, machine learning, user study

## Abstract

Generating spatial referring expressions is key to allowing robots to communicate with people in an environment. The focus of most algorithms for generation is to create a non-ambiguous description, and how best to deal with the combination explosion this can create in a complex environment. However, this is not how people naturally communicate. Humans tend to give an under-specified description and then rely on a strategy of repair to reduce the number of possible locations or objects until the correct one is identified, what we refer to here as a dynamic description. We present here a method for generating these dynamic descriptions for Human Robot Interaction, using machine learning to generate repair statements. We also present a study with 61 participants in a task on object placement. This task was presented in a 2D environment that favored a non-ambiguous description. In this study we demonstrate that our dynamic method of communication can be more efficient for people to identify a location compared to one that is non-ambiguous.

## 1. Introduction

The ability to generate Spatial Referring Expressions is a key requirement to allow robots to communicate naturally with people within an environment. Spatial Referring Expressions are when we use spatial language to identify an object or describe a location, for example “You left your keys under the folder on the desk” (Viethen and Dale, 2008). Such expressions are used commonly by people when identifying an object, even if another property—such as color—of the object could be used to create a unique description. Understanding and producing these descriptions can be useful for an interactive agent, such as Viethen's example as an assistive domestic agent, or to direct an embodied robot in a navigational task (Tellex et al., 2011).

A typical assumption in robot development is that the best description is one that allows an object or location to be uniquely described (Dale and Reiter, 1995). We refer to these descriptions in this paper as non-ambiguous as such a description leaves no room for an object or location to be mistaken with another. These descriptions take a very rigid approach to the Gricean Maxims (Grice, 1975):

- *Maxim of Quantity*: The referring expression should provide the relevant information without extra un-required information.
- *Maxim of Quality*: The referring expression should be true.
- *Maxim of Relation*: The referring expression should be relevant.
- *Maxim of Manner*: The referring expression should be clear as to its contribution, timely and avoid ambiguity.

However, other than the maxim of quality Grice states that the other maxims may vary in importance. These approaches also have an issue of combinatorial explosion when attempting to generate expressions in a large problem space. More recent algorithms attempt to reduce this problem space as much as possible. One example uses landmarks to narrow down the list of objects that could be referred to Kelleher and Kruijff (2006). Taking into account the potential knowledge of a human interactant also reduces the problem, as computations are only needed on what a person is aware of Ros et al. (2010) and Lemaignan et al. (2012). This also makes for a description that is more useful to the listener.

Evaluation frameworks for generation algorithms often have a bias toward a complete and non-ambiguous description by being based upon a single direction of communication (Williams and Scheutz, 2017). This highlights the fact that we often take the description as the goal, and not the communication that a description is for Krahmer and Van Deemter (2012). Communicating the location of an object to someone else is often a two way communication (Clark and Wilkes-Gibbs, 1986). Between people, descriptions are often under-specified and a dynamic strategy of repair is used to correct these mistakes. This strategy allows for the sharing of cognitive load between a describer and a listener. Each participant is able to contribute to the discourse until a grounding criterion is met (Clark and Schaefer, 1989). It is only when there is difficulty reaching alignment that a full description becomes necessary (Pickering and Garrod, 2006). In children this process can be highly dynamic with the child receiving the description making actions to prompt the child in the role of describer, or allow for a simpler description (Wallbridge et al., 2018).

Work on understanding a referring expression realizes that the description provided by a person is often ambiguous and steps need to be taken to disambiguate it (Shridhar and Hsu, 2018). This process can be cumbersome for a robot, with a lot of dialogue required to narrow down a description, often relying on confirmation every step of the way, as with installment descriptions (Fang et al., 2015). However, much of the information can be disambiguated by the situational context (Magassouba et al., 2018), or by allowing an agent to realize that a single command may require multiple action steps (Tellex et al., 2011).

The use of a dynamic description given by a robot should be investigated for potential benefits. However, this area of research (ambiguous spatial referring) has seen little research by the community. While the interactions between two people are often dynamic in a normal interaction, we want to explore if that would be beneficial in the case of interactions between a robot and a human. Such descriptions may be less stilted and more natural in their presentation, and more efficient in helping a person narrow down the referent in an interaction.

Here we present our method for generating dynamic descriptions for spatial referring expressions (section 2.4). We also present a study in which we compare our generation of dynamic descriptions against a non-ambiguous system. The systems are compared on efficiency by looking at time to complete a task of placing objects in the correct positions, and on people's preference with the use of a questionnaire.

## 2. Methodology

### 2.1. Research Question

As well as seeing if it is possible for us to generate an *ad-hoc* dynamic spatial description, we wanted to test that it would be an effective method for a robot to use when describing spatial locations to people. We wanted to design a task that made it easy to generate scenarios that require non-ambiguous descriptions of varying complexity. This would also mean the task would favor a non-ambiguous description, making it more conclusive if an ambiguous description were able to surpass its performance. We thought it would also be important to test people's preferences, as there is potential for a difference in our expectations between a robot and a person (Malle et al., 2015).

### 2.2. The Task

**Figure 1:**
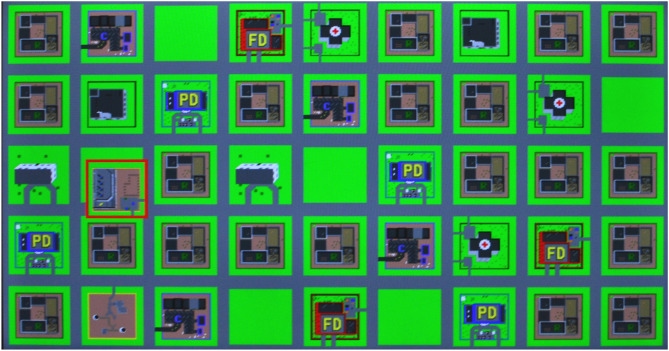
An example of the game in progress. The object with the red border is being moved to one of the empty spaces based on a description like “the power plant is below a church”.

All the interactions for our study were based around a “city planning” game. In this game a participant had to move the picture of a building to an empty location on a map. These locations can only be described with confusing, ambiguous, or complex utterances (requiring between 1 and 4 descriptors to create a non-ambiguous statement. For example a non-ambiguous statement requiring two descriptors would be “A residence is above a commercial district and to the right of a fire department” (see Figure 1). Models for our game were taken from Micropolis^1^, an open source version of the original Sim City. The game itself was presented on a tabletop touchscreen, which we call the “sandtray” (Baxter et al., 2012).

The game started with a tutorial screen that showed all the 9 buildings that were used with descriptions that could be—and in the case of the robot describing, were—used to identify them. The game itself had two rounds, each with a different map. Both maps had 12 empty spaces and 26 objects already in position. Each round required the placement of 7 objects to complete. Each round had 2 objects that required 1 descriptor to be un-ambiguously placed, 2 that required 2 descriptors, 2 that required 3 descriptors, and 1 that required 4 descriptors. These buildings were shown one at a time for placement and were highlighted with a red border to show that they could be moved. These objects needed to be touched and dragged into position. The checking of the position would only occur after a short delay when the object had been released. This was to prevent an over reliance just trying to guess the position, or by swiping the objects through each empty space.

### 2.3. Hypotheses

We designed our study to test the following hypotheses:

H1: A robot giving a dynamic description (an ambiguous initial statement with follow up repair) would allow a person to more quickly locate the correct placement of objects than when a robot is giving a non-ambiguous description.
H2: A robot giving a dynamic description would be preferable for a person compared to a robot giving a non-ambiguous description.

### 2.4. Implementation

We used Underworlds (Lemaignan et al., 2018) to represent the state of the world. Underworlds is a software solution that enables us to represent both a real and virtual environment with meshes, enabling us to reason about the location and relations of objects. While our game is a 2D interaction Underworlds represents the state of the game in 3D, allowing for extending this system to 3D situations in future work. Spatial relations could be calculated with the use of bounding boxes to identify nearby objects as landmarks (Kelleher and Kruijff, 2006) and perform relatively simple geometric comparisons. From these spatial affordances we would then build a natural language description. In the case of generating a non-ambiguous description, in a similar fashion to the Incremental algorithm (Dale and Reiter, 1995), we then add descriptors in a greedy fashion that remove ambiguity until none remained. Descriptions were repeated every 5 s after the robot had finished speaking until completion of the placement.

For the dynamic description we wanted to have a fully automated method of providing repair—following an initial statement selected from one of the generated affordances—that was based on interactions between two people. With this in mind we conducted a series of pre-studies for data acquisition, using the game described in section 2.2. The first pre-study (*N* = 18) was an interaction between two people. The participants would after a round switch roles; one would be describer—the describer has a map showing the desired locations of the buildings—and one would be the manipulator—who moves the objects based on the describer's description. From this interaction we gathered the coordinates of the object as they were moved by the participants, at a rate of 10 Hz. Taking these coordinates we calculated the distance to target, change in distance to target, magnitude of motion and change in angle from previous sample of motion to represent the state of interaction. We would then annotate the type of feedback provided by the person in relation to that motion, and label the interactions states with that feedback category. We used the following categories for the annotation:

- *Negate*- A negative response indicating that the manipulator is heading in the wrong direction (e.g., “No.”).
- *Elaborate*- A response to give more information, when the manipulator appears to be hesitating (e.g., “…and to the left of the hospital.”).
- *Positive*- A positive response given to the manipulator to indicate they are heading in the right direction (e.g., “Yes.”).

Two subsequent pre-studies (*N* = 8, *N* = 9) tested the non-ambiguous condition and a prototype version of the dynamic that used a SVC classifier (Wallbridge et al., 2019). Data from these studies was used to augment data collected from the first pre-study. This gave us 5230 sample states with annotations of which 4159 (~80%) were randomly selected for training a classifier and the remainder were used for testing.

Our prototype version of the dynamic condition struggled with correctly classifying data that should result in a negate classification, often assigning them as elaborate. This was in part due to the relatively small number of records classified as negate compared to the others. Even with the additional data we collected from the subsequent studies there was still a high amount of confusion for this category. Therefore, we looked for an alternative method. For the classifier used in the dynamic condition in this study we trained an MLP network^2^. This network used 3 fully connected hidden layers, each of size 20, a ReLU activation and an LBFGS solver. From this we obtained a 96.5% success rate. The confusion matrix can be seen in Table 1.

**Table 1:** Confusion matrix of the MLP classifier used for the dynamic condition.

		**Prediction**		
		**Negate**	**Elaborate**	**Positive**
Actual	Negate	182	4	0
	Elaborate	4	397	19
	Positive	0	10	455

*The data used to train the MLP was obtained from a series of pilot studies*.

When trying to get natural timing for feedback our data showed a huge variance. We believe that this is due to the person in the role of describer trying to process an environment with which they are unfamiliar, and making their own mistakes without realizing. Therefore, in this study we decided to use a manually coded timing mechanism. Feedback was based on an average result from the classifier over the previous half a second—still sampled at 10 Hz. In the case of a negate statement being required the robot would respond immediately. The robot would only give a positive statement if it hadn't spoken in the previous 2 s. An elaborate statement would be given if the robot had not spoken for 1 s if the information was different to the previous. Otherwise the robot would repeat the previous statement after 5 s. Future work may emphasize establishing more natural timing and amount of feedback.

The dynamic description starts by giving an initial statement of a single descriptor that would disambiguate from at least some of the potential locations. From there the classifier takes over based on the user's actions. In the case of a negate statement being required either “no” or “nope” was randomly selected. In the case of Positive feedback either “yes”, “right”, or “yup” was randomly selected. When an elaborate statement was required, a descriptor that would disambiguate from the current location to the target location would be given.

### 2.5. Questionnaire

In order to test H2 we used a questionnaire. The questionnaire was split into 3 parts that would be administered after being introduced to the robot, upon completion of the first round, and at the end of the study after the second round.

The initial part of the questionnaire included details on demographics. In all 3 sections we asked the same questions that were taken from the Godspeed questionnaire (Bartneck et al., 2009) to give quantitative measurements:

- *Anthropomorphism*: Fake to Natural, Machinelike to Humanlike, Unconscious to Conscious, and Artificial to Lifelike.
- *Likeability*: Dislike to Like, Unfriendly to Friendly, Unpleasant to Pleasant, and Awful to Nice.
- *Perceived Intelligence*: Incompetent to Competent, Ignorant to Knowledgeable, Unintelligent to Intelligent, Foolish to Sensible, and Inert to Interactive.

After each round we also asked questions with a Likert scale on how helpful they found the robot, whether they found feedback timely and if it was appropriate. We also asked them about the amount of feedback they received from the robot on whether it was too little to too much.

At the end of the last part of the questionnaire we also asked the participants several open ended questions. The first was to describe the difference between the behavior of the robot in each round. The second question directly asked the participants for their preference and their reason. The third question asked them to describe any behaviors of the robot other than its description that aided in the completion of the task. We also left a section for any other comments the participants wanted to make.

### 2.6. Interaction

A within subject design was used, with the order of the conditions (dynamic and non-ambiguous) counter-balanced across participants. The order of conditions was randomly assigned to the next participant before the experimenter would meet them. The order of the map was also controlled, the first 40 participants saw map 1 and then map 2, whereas for the remainder of participants this was reversed.

**Figure 2:**
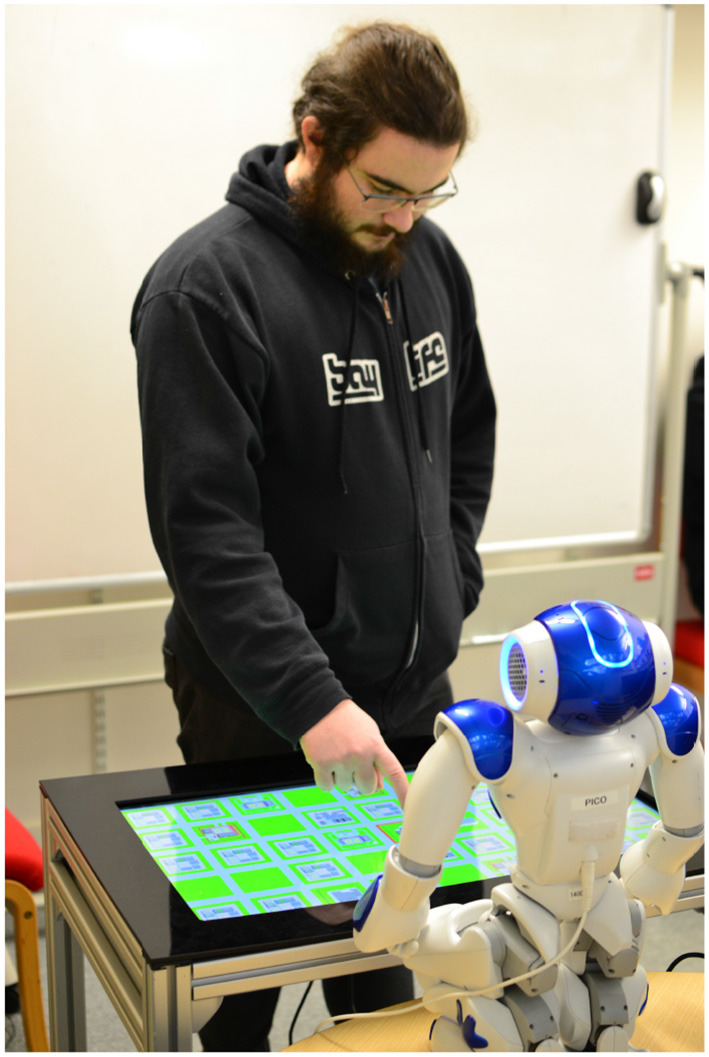
A participant interacting in our study. The robot kneels on the opposite side of a touch screen from the participant. The robot provides descriptions that allows the participant to use the touch screen to drag and place a building on the map. Written consent was provided for the use of this image.

Upon entering the room the participant was introduced to the robot, a Softbank Nao. The robot at this point was in a crouching position with a slight breathing animation, and performing face tracking. The participant was then asked to complete the first part of the questionnaire. The participant was then directed to stand opposite the robot (see Figure 2). The experimenter would give a few initial details. The experimenter told the participant that the robot would give instructions on how to play the game, and explained the purpose of a secondary screen—positioned so that actions on the touch screen were visible to the camera recording the interaction and to the experimenter observing—so that they were aware their actions were being observed and recorded.

The game proceeded as described in section 2.2, with the robot explaining the game to participants. In both rounds the robot acted as describer to the participants. In both conditions the robot would also look at the target while describing, and then back at the participant between descriptions. It was believed that this looking behavior would provide some contextual information to the participant, while still requiring a verbal description to narrow down the location. Based on the condition order randomly assigned to the participant they would see either the dynamic or non-ambiguous condition during the first round. We treated the first object in each round as a practice question—in both maps this was an object that required 1 descriptor—and the experimenter would repeat instructions if required to the participants.

After round 1 participants were asked to complete the second part of the questionnaire. The participant was informed that while they were filling out the questionnaire that the experimenter was changing the behavior of the robot, which the experimenter would pretend to do. Having filled in the questionnaire the participant proceeded to stand opposite the robot again. The experimenter informed the participant that the game would be exactly the same as before, but re-iterated that the robot's behavior had now changed. This was to ensure that our manipulation of the robot's behavior was perceived.

Round 2 proceeded the same as the first with a different map, and with the condition that the participant had yet to see. The final part of the questionnaire was administered before participants were then debriefed on the study. The entire interaction—including completing the questionnaire—typically lasted 10–15 min. Participants were paid a £5 Amazon gift voucher upon conclusion of their participation for their time.

Data (position of the target and of the moving object, completion time, and video recording) was recorded on all interactions. An example of a transcribed log of the interaction can be found in Table S1 of the Supplementary Materials.

### 2.7. Demographics

For this study we recruited 61 participants (33 female) from the vicinity of the University of Plymouth Campus, meaning the majority of our participants were students of varying disciplines. Participants had a mean age of 23.8 years (min = 18, max = 64, *sd* = 9.16). Three participants did not speak English as their first language, but their fluency was judged to be high enough to not require excluding them from the study. Twenty-two participants (13 female) described themselves as having had no previous interaction with robots. Thirty-three participants (17 female) described themselves as having had previous interactions with commercial robots. Three participants (2 female) said they worked alongside robots. Three participants (1 female) described themselves as robotocists. Thirty-one participants (14 female) saw the non-ambiguous condition followed by the dynamic, with the remaining 30 (19 female) seeing the dynamic condition first.

## 3. Results

### 3.1. Task Performance

**Figure 3:**
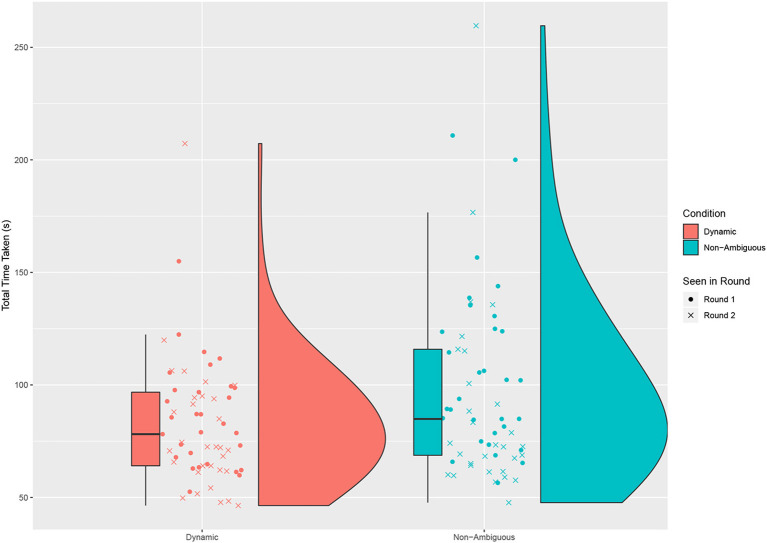
The sum of time taken to complete all the placements in a round. For each placement the time is taken from the moment the robot starts the first description to the moment the object is successfully placed. There is a significant difference between the time taken in the dynamic Condition and non-ambiguous condition when both Round 1 and Round 2 are considered together.

To start measuring task performance we looked at the difference in completion time across all questions (excluding the first practice question) in a round, split by condition. For each object after the first we would take the time from when the robot started speaking, to the moment that the object was correctly placed. No significant difference is seen in the completion times for each round when the order the maps are presented in is changed, suggesting no order effect [Welch Two Sample *t*-test: Round 1−*t* = 0.161, *df* = 50.184, *p* = 0.873, mean of Map 1 = 96.4 s (*sd* = 34.8), mean of Map 2 = 95.1 s (*sd* = 27.2). Round 2−*t* = −1.262, *df* = 58.749, *p* = 0.212, mean of Map 1 = 77.0 s (*sd* = 23.9), mean of Map 2 = 87.8 s (*sd* = 43.1)]. As such we do not continue to treat this data separately. We found a significant difference between the dynamic and non-ambiguous conditions [Paired *t*-test: *t* = −2.202, *df* = 60, *p* = 0.032, mean of dynamic = 82.9 s (*sd* = 27.1), mean of non-ambiguous = 97.2 s (*sd* = 41.1); see Figure 3].

**Figure 4:**
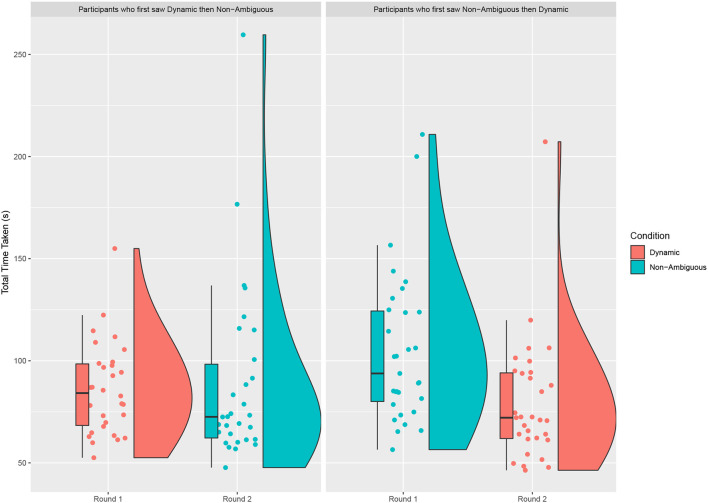
The sum of time taken to complete all the questions on the map by the order seen. For each question the time is taken from the moment the robot starts the first description to the moment the object is successfully placed. Independent of the order seen there is a clear training effect between round 1 and round 2. When those who saw the dynamic condition in Round 1 is compared to those who saw the non-ambiguous condition we see a significant difference. However, when we compare those who saw the dynamic condition in round 2 against those who saw the non-ambiguous condition we see no significant difference.

However, we also see a significant practice effect when we look at the completion time for round 1 compared to round 2 [Paired *t*-test: *t* = 2.175, *df* = 60, *p* = 0.034, mean of Round 1 = 96.0 s (*sd* = 32.1), mean of Round 2 = 84.1 s (*sd* = 37.8)]. So we decided to take a look at the difference between conditions across rounds (see Figure 4). When those who saw the dynamic condition in round 1 are compared to those who saw the non-ambiguous condition we see a significant difference [Welch Two Sample *t*-test: *t* = −2.435, *df* = 49.656, *p* = 0.019, mean of dynamic = 86.2 s (*sd* = 22.6), mean of non-ambiguous = 105.4 s (*sd* = 37.3)]. However, when we compare those who saw the dynamic condition in round 2 against those who saw the non-ambiguous condition we see no significant difference [Welch Two Sample *t*-test: *t* = −0.944, *df* = 51.969, *p* = 0.349, mean of dynamic = 79.6 s (*sd* = 30.9), mean of non-ambiguous = 88.8 s (*sd* = 43.8)].

**Figure 5:**
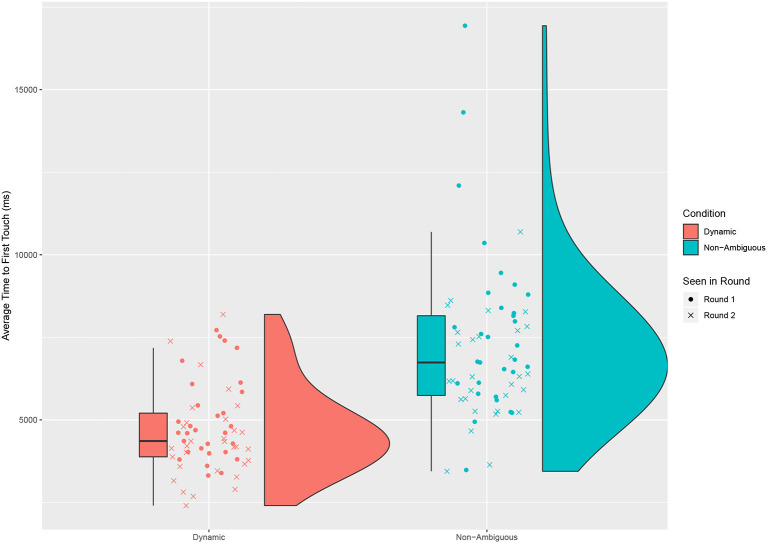
The average time taken for a participant to first touch the object across all the questions in a round. For each question the time is taken from the moment the robot starts the first description to the moment the participant first touches the object. A significant difference is found between the dynamic and non-ambiguous conditions.

We look at several factors to try and explain some of the differences in time taken. The first was how long it would take the participant to act, by looking at how long it took a participant to first touch an object after the description began (see Figure 5). We saw a significant difference across conditions [Paired *t*-test: *t* = −7.449, *df* = 60, *p* < 0.001, mean of dynamic 4,707 ms (*sd* = 1317), mean of non-ambiguous = 7,157 ms (*sd* = 2308)].

**Figure 6:**
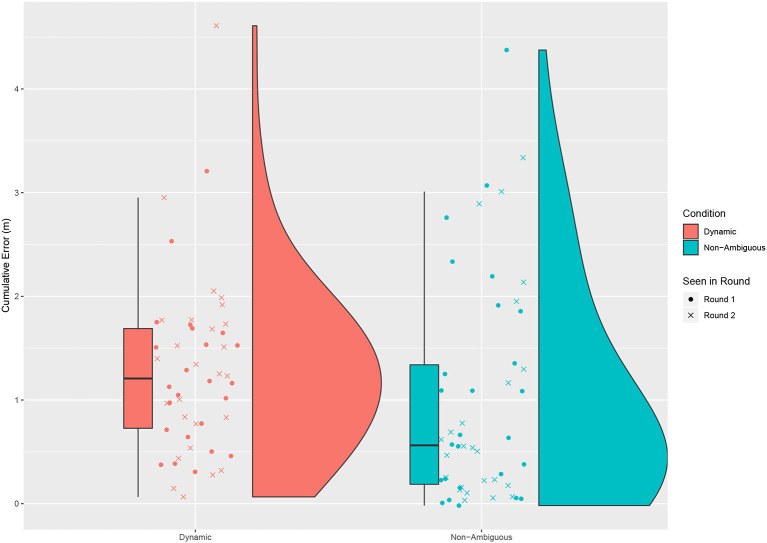
The cumulative error in distance an object was moved beyond the required straight line distance required to the correct answer for an entire round. No significant difference was found between the dynamic and non-ambiguous conditions.

We also analyzed the error in placement by looking at the distance the object was moved compared to the required direct distance to reach the target cumulatively across all placements for a round (see Figure 6). Unfortunately due to a technical issue some of the recordings for touch data failed to be recorded, as such 11 participants were not used in this analysis, leaving us with 50 participants (24 dynamic First). While we would expect to see a greater error in the dynamic condition there was no significant difference between the two conditions [Paired *t*-test: *t* = 1.790, *df* = 60, *p* = 0.079, mean of dynamic = 0.987 m (*sd* = 1.148), mean of non-ambiguous = 0.597 m (*sd* = 1.285)].

### 3.2. Preference

**Figure 7:**
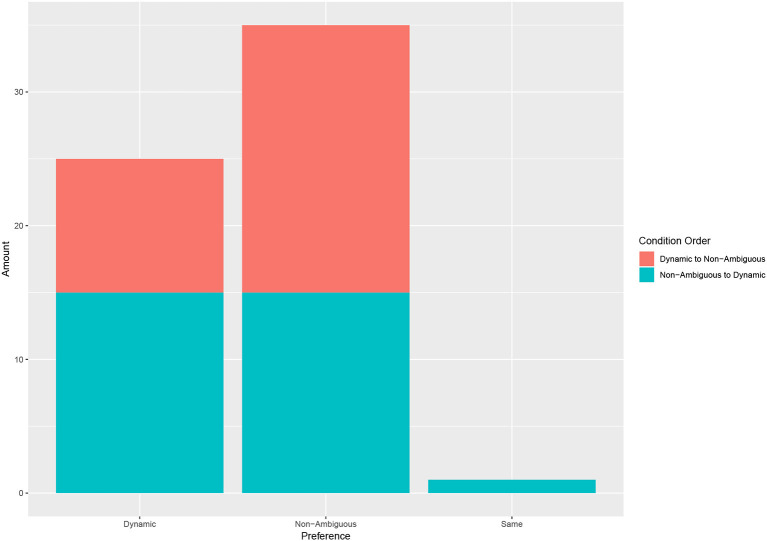
The stated preference of participants of each condition. The condition order does not have a significant effect on the stated preference.

A first look at the participant's stated preference shows a preference toward the non-ambiguous condition (see Figure 7) with 25 participants stating they preferred the dynamic, 35 answering non-ambiguous and 1 who did not have a preference. Note that we saw no significant difference caused by the order in which the conditions were seen (Fisher's Exact Test: *p* = 0.243).

**Figure 8:**
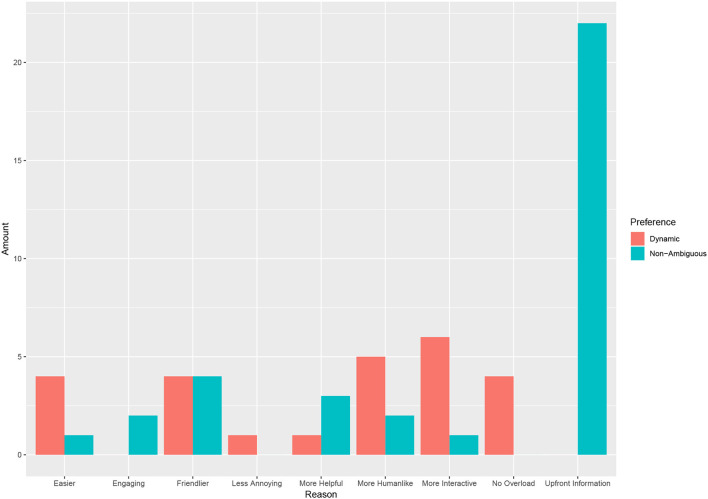
The stated reason of preference by condition. Note that the reason was an open ended question and were categorized by the authors.

From the open ended answers given for preference we split these into categories. Looking at these categories (see Figure 8) we see a variety of reasons given for preferring the dynamic condition. Overwhelmingly though, the stated reason for liking the non-ambiguous condition was that participants preferred having all the information upfront.

Twenty-seven participants stated that they found the head movement looking at the target helpful (14 saw dynamic first). However, only 9 of these participants stated they preferred the dynamic condition.

For further analysis of the quantitative data 5 participants were not considered as they failed to answer some of the questions leaving us with 56 participants (28 dynamic First). Due to issues with participants committing to their first answers in the questionnaire we chose to do a between subject analysis based on their responses after the first round. Distributions were normal. In the category of Anthropomorphism we saw that the dynamic condition was considered significantly more humanlike [Welch Two Sample *t*-test: *t* = 2.158, *df* = 52.563, *p* = 0.035, mean of dynamic = 3.036 (*sd* = 0.962), mean of non-ambiguous = 2.429 (*sd* = 1.136)] and more conscious [Welch Two Sample *t*-test: *t* = 2.149, *df* = 53.712, *p* = 0.036, mean of dynamic = 3.607 (*sd* = 1.257), mean of non-ambiguous = 2.857 (*sd* = 1.353)].

In the category of perceived intelligence we see that the dynamic condition was considered significantly more interactive than the non-ambiguous [Welch Two Sample *t*-test: *t* = 3.093, *df* = 50.638, *p* = 0.003, mean of dynamic = 4.179 (*sd* = 1.353), mean of non-ambiguous = 3.393 (*sd* = 1.257)].

No significant differences were found in the category of likeability. No significant difference was found in how helpful they found the robot to completing the task [Welch Two Sample *t*-test: *t* = 1.536, *df* = 53.277, *p* = 0.131, mean of dynamic = 4.000 (*sd* = 0.981), mean of non-ambiguous = 3.571 (*sd* = 1.103)]. No significant difference was found in the case of how appropriate the feedback was [Welch Two Sample *t*-test: *t* = –0.142, *df* = 51.101, *p* = 0.887, mean of dynamic = 4.143 (*sd* = 1.044), mean of non-ambiguous = 4.179 (*sd* = 0.819)]. No significant difference was found on timeliness of feedback [Welch Two Sample *t*-test: *t* = 0.384, *df* = 53.883, *p* = 0.703, mean of dynamic = 3.679 (*sd* = 1.020), mean of non-ambiguous = 3.571 (*sd* = 1.069)]. No significant difference was found in the amount of feedback [Welch Two Sample *t*-test: *t* = –1.443, *df* = 48.993, *p* = 0.155, mean of dynamic = 2.714 (*sd* = 0.535), mean of non-ambiguous = 2.964 (*sd* = 0.744)].

## 4. Discussion

Overall we can see a better performance of the dynamic condition vs. the non-ambiguous in this task. However, it would seem that this is only truly advantageous when beginning an unfamiliar task. While we did not measure the cognitive load, this could be explained as due to a higher cognitive load while learning the task, and the dynamic condition helps to reduce this. Future work may look at tasks where the cognitive load remains high, to see if the advantages of the dynamic condition may remain over a longer term.

An obvious advantage to the dynamic description is that by having a shorter description to listen to, people can start to act faster. We also see a much greater distribution in action time across the non-ambiguous condition, which again may be attributed to a higher cognitive load, as some participants required longer to think about the answer.

An unexpected result was the lack of a significant difference on the distance errors by participants. While descriptions should have been fully non-ambiguous it is worth noting that if a person forgot what name was assigned to a building that the description would now be ambiguous to them, causing these errors. It would also again be worth looking at cognitive load to see how many cases were caused by too much information given at once. Looking at the distribution a large number of users made almost no error in the non-ambiguous condition. However, we see a greater spread in those who were struggling by making more mistakes. Therefore, we believe that even when giving what is believed to be a completely non-ambiguous description that it is advantageous to be able to produce repair statements. The fact that the distance error in the dynamic condition is equal or greater means we are seeing people moving and correcting mistakes faster. We should however consider that the physical task space was relatively small, and that in a larger task space mistakes may be more costly. Still this may be mitigated by how quickly we can identify when a person is choosing the wrong target.

While we see some increase in anthropomorphism and interactivity with the dynamic condition over the non-ambiguous, this did not translate into a stated preference for the dynamic condition, so H2 is not supported. We can also only state partial support for $\bar{\text{H2}}$ as we find no significant results in Likeability. Several factors of our implementation may have contributed to a preference for the non-ambiguous as well. Firstly the TTS generation of the robot's negative feedback was very harsh and petulant. This often caused the robot to be perceived as critical or rude during the dynamic condition by the participant, leading them to believe it was less friendly. Secondly our task was short and game-like, and we did not inform participants by what metrics they would be judged prior to starting the experiment. This led to many participants valuing accuracy higher than efficiency. A longer and more real world scenario may lead participants perception to shift toward a preference for efficiency.

The game-like nature of our tasks makes for a simple comparison between non-ambiguous and dynamic descriptions. Our non-ambiguous algorithm can be fairly simplistic as we are not currently concerning ourselves with different perception models as in a more complex algorithms (Fang et al., 2013). This also means a simple non-ambiguous description exists. However, more complex environments are more likely to be encountered in real world situations, making generating a non-ambiguous description much more complicated, and complex to understand. A dynamic description can simplify this process between human and robot, no longer requiring full cognitive alignment, rather we only need to be able to provide some descriptors for our target, and then be able to disambiguate from an incorrect target.

One potential avenue of further investigation is to look at an interactant's ability in spatial tasks and memory. Participants' preference for upfront information would likely be affected by their proficiency at the task. At some point however, a description must become too complex for any person, at which point installments of information becomes necessary. It would be practical for the robot to be able to adjust the amount of upfront information provided before proceeding to contextual repair statements. Potentially this could be trained by looking at the amount of hesitation before action, and confidence of action (speed and directness). We may also consider that in the short duration of our study we may miss that after further practice that more up-front descriptions become more beneficial as the task continues.

## 5. Conclusions

We presented a method by which a robot can generate dynamic spatial referring expressions to describe a location. We did this using a system that can generate some simple disambiguating statements, and then provided repair statements based on an interactant's actions. To do this we trained an MLP network from human-human data that looked at the distance to the target, the change in distance, the magnitude of motion, and the change in angle as participants moved an object.

We found that using a dynamic system of partial information with follow up repair statements was a more efficient method of describing a location when the task is unfamiliar than by just giving a non-ambiguous statement—partially supporting our first hypothesis. However, this advantage was not found in a subsequent round of our task, suggesting that this may be due to the short amount of practice given, and the discovery required in a new task. This may contribute to an increased cognitive load during the first round. Future work should attempt to measure the cognitive load, and if this advantage may be due to a higher load, or specifically when a task remains unfamiliar to a person.

While our dynamic system implementation showed benefits in perception of being more humanlike and being more interactive, participants stated preference was for the non-ambiguous condition. But no significant difference was found in likeability. Therefore, for this implementation we reject our second hypothesis and suggest partial support for the opposite. Our analysis shows that a major reason for this is a preference for upfront information. This may be in part due to our task being game-like and the interaction short. However, improvements could be made to the dynamic description by adaptively adjusting how much upfront information is given based on user interactions. With this in mind further work should also look at an interaction that lasts longer, to see if more upfront information may continue to grow more advantageous as time goes on.

Our implementation was restricted to a game-like scenario on a touch screen, where all objects were easily visible to both the robot and the participant. Future work should focus on an implementation in a more real world scenario, including issues of perspective and occlusion.

Even when a robot provides a completely non-ambiguous spatial description people are liable to make errors when trying to locate an object or target. Therefore, robots should be prepared to provide more dynamic repair statements to aid in correcting mistakes as efficiently as possible.

## Data Availability

The datasets generated for this study are available on request to the corresponding author.

## Ethics Statement

This study was carried out in accordance with the recommendations of the Plymouth University Faculty of Science and Engineering Research Ethics Committee with written informed consent from all subjects. All subjects gave written informed consent in accordance with the Declaration of Helsinki. The protocol was approved by the Plymouth University Faculty of Science and Engineering Research Ethics Committee. No animals or vulnerable populations were involved in this study.

## Author Contributions

CW, SL, and TB contributed conception of study. CW, SL, and ES contributed to design of the study. CW performed study, performed the statistical analysis, and wrote the first draft of the manuscript. ES made major modifications to sections of the manuscript. TB obtained funding for the research. All authors contributed to manuscript revision, read, and approved the submitted version.

### Conflict of Interest Statement

The authors declare that the research was conducted in the absence of any commercial or financial relationships that could be construed as a potential conflict of interest.

## Footnotes

^1^https://github.com/SimHacker/micropolis

^2^This network was trained using the scikit-learn library for Python (Pedregosa et al., 2011).
